# Epigenetics and Regulation of Oxidative Stress in Diabetic Retinopathy

**DOI:** 10.1167/iovs.18-24548

**Published:** 2018-10

**Authors:** Arul J. Duraisamy, Manish Mishra, Anjaneyulu Kowluru, Renu A. Kowluru

**Affiliations:** 1 Department of Ophthalmology, Wayne State University, Detroit, Michigan, United States; 2 Pharmaceutical Sciences, Wayne State University, Detroit, Michigan, United States; 3 John D. Dingell VA Medical Center, Detroit, Michigan, United States; 4 Anatomy/Cell Biology, Wayne State University, Detroit, Michigan, United States

**Keywords:** diabetic retinopathy, DNA methylation, epigenetics, oxidative stress, ras-related C3 botulinum toxin substrate 1 (Rac1)

## Abstract

**Purpose:**

Oxidative stress plays a central role in the development of diabetic retinopathy, and in the pathogenesis of this blinding disease, activation of NADPH oxidase 2 (Nox2)-mediated cytosolic reactive oxygen species (ROS) production precedes mitochondrial damage. The multicomponent cytosolic Nox2 has an obligatory component, Ras-related C3 botulinum toxin substrate 1 (Rac1); in diabetes, Rac1 is functionally and transcriptionally active. Diabetes also facilitates many epigenetic modifications, and activates both DNA methylating (Dnmts) and hydroxymethylating (Tets) enzymes. Our aim was to investigate the role of epigenetics in *Rac1* regulation in diabetes.

**Methods:**

Using human retinal endothelial cells, exposed to high glucose, 5-methyl cytosine (5mC) and 5-hydroxy methyl cytosine (5hmC) levels, and binding of Dnmt and Tets were quantified at the *Rac1* promoter. The effect of inhibition of Dnmts/Tets (pharmacological inhibitors or short interfering RNA [siRNA]) on glucose-induced activation of Rac1-ROS production was evaluated. Results were confirmed in retinal microvessels from streptozotocin-induced diabetic mice receiving intravitreally *Dnmt1*-siRNA.

**Results:**

Despite high glucose-induced increased binding of Dnmt1, 5mC levels remained subnormal at *Rac1* promoter. But, at the same site, 5hmC levels and transcription factor nuclear factor (NF)-*k*B binding were increased. Inhibition of Dnmts/Tets prevented increase in 5hmC and NF-*k*B binding, and attenuated Rac1 activation. Similarly, in mouse retinal microvessels, *Dnmt1*-siRNA ameliorated diabetes-induced increase in *Rac1* transcripts and activity, and decreased ROS levels.

**Conclusions:**

Thus, despite Dnmts activation, concomitant increase in Tets rapidly hydroxymethylates 5mC, allowing NF-κB to bind and activate *Rac1*. These results imply a critical role of an active DNA methylation in cytosolic ROS regulation in the development of diabetic retinopathy.

Diabetic retinopathy, a slow progressing complication, is the leading cause of blindness in working age adults. The pathology of the disease is complex and involves many interrelated metabolic abnormalities initiated by persistent high circulating glucose.^1^ Increase in cytosolic reactive oxygen species (ROS) in the retina and its vasculature is an early event in the development of diabetic retinopathy.^2^ In the cytosol, ROS are mainly generated by NADPH oxidases (Nox), and among this family of enzymes, Nox2 is one of the isomers activated in the retina in diabetes.^3^ Nox2 is a multicomponent enzyme, and for its stabilization, a small molecular weight GTPase, Ras-related C3 botulinum toxin substrate 1 (Rac1),^4^ plays an important role. Experimental models have shown that the activation of Rac1-Nox2-ROS precedes mitochondrial damage, and the development of diabetic retinopathy.^5^

Functional activation of Rac1 requires specific guanine exchange factors, including Tiam1 and Sos1, and in diabetes, Tiam1-mediated activation of Rac1 is implicated in retinal Nox2-ROS production.^2,5^ Hyperglycemic milieu also increases *Rac1* transcription, and our recent work has shown that hyperglycemia promotes this transcriptional activation by increasing the binding of nuclear transcriptional factor-κB (NF-κB, p65 subunit) at the *Rac1* promoter.^6^ However, the putative mechanism underlying increased transcription factor binding remains to be explored.

Epigenetic modifications, the modifications that can alter gene expression without affecting the DNA sequence, play an important role in regulating the binding of the transcription factors in many disease conditions.^7^ Many epigenetic modifications, including DNA methylation and histone acetylation, are shown to play a role in the development of diabetic complications, including retinopathy.^8–12^ Addition of a methyl group to the cytosine by DNA methyl transferases (Dnmts) forms 5-methylcytosine (5mC), and methylated cytosine impedes transcriptional factor binding, resulting in gene repression. However, 5mC can be rapidly oxidized by Ten-Eleven Translocation dioxygenases (Tets), and the resulting formation of 5-hydroxymethylcytosine (5hmC) stimulates gene expression.^13–16^ In diabetes, both Dnmts and Tets are activated in the retina and its vasculature, and among these two families of enzymes, Dnmt1 and Tet2 are the only respective isoforms that are upregulated.^9^ How these opposing enzymes regulate DNA methylation status of *Rac1* is not clear.

The aim of this study was to investigate the role of epigenetic modifications in the transcriptional regulation of *Rac1*. Using human retinal endothelial cells (HRECs), we investigated the effect of high glucose on DNA methylation status of the *Rac1* promoter. The role of DNA methylation in *Rac1* transcriptional activation was further established using pharmacological inhibitors and specific small interfering RNAs (siRNAs) of *Dnmt1* and *Tet2*. Key parameters were confirmed in an in vivo model using retinal microvessels from streptozotocin-induced diabetic mice, receiving intravitreal administration of *Dnmt1*-siRNA or negative control siRNA.

## Methods

### Retinal Endothelial Cells

**Figure 1:**
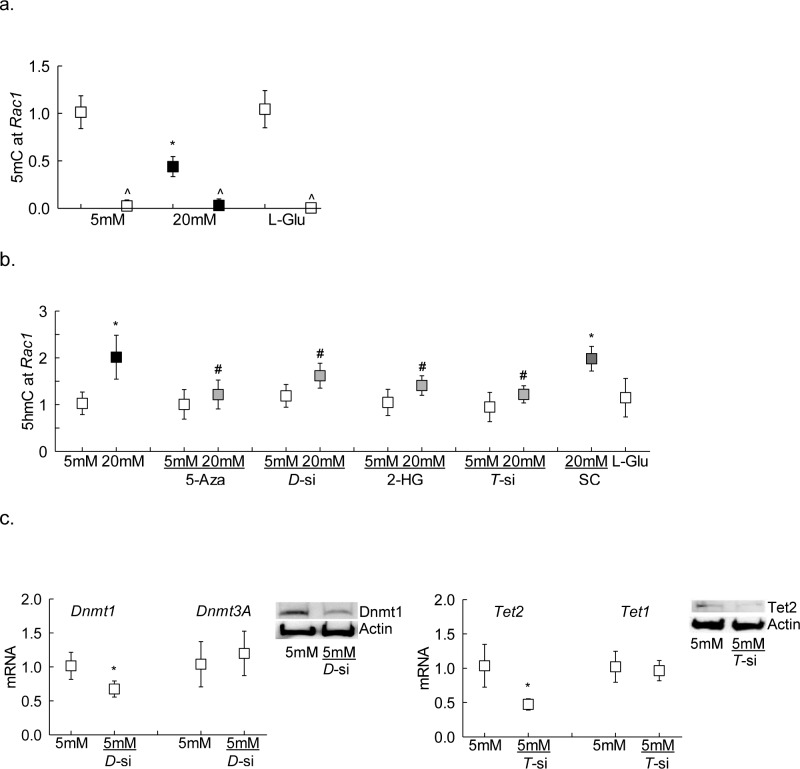
Effect of high glucose on Rac1 promoter DNA methylation. (a) Levels of 5mC and (b) 5hmC were quantified at Rac1 promoter in HRECs incubated in high glucose for 96 hours by immunoprecipitating genomic DNA with their respective antibodies. IgG (^) was used as an antibody control. Gene expressions of (c) Dnmt1 and Dnmt3a, and (d) Tet2 and Tet1 were measured by qRT-PCR using β-actin as a housekeeping gene, and protein expressions of Dnmt1 and Tet2 were determined by Western blot technique using β-actin as a loading protein. Values obtained from HRECs in 5-mM glucose are considered as 1, and are represented as mean ± SD from three different cell preparations, with each measurement performed in duplicate: 5 mM and 20 mM indicate 5-mM and 20-mM glucose, respectively; L-Glu indicates 20-mM L-glucose; 5-Aza and 2-HG indicate cells incubated with 5-Aza or 2-HG; D-si, T-si, and SC indicate Dnmt1-siRNA or Tet2-siRNA or scrambled control RNA, respectively. *P < 0.05 compared with 5-mM glucose; #P < 0.05 compared with 20-mM glucose.

HRECs, obtained from Cell Systems Corporation (catalog no. ACBRI 181; Cell Systems Corp., Kirkland, WA, USA), were cultured in Dulbecco's modified Eagle's medium (DMEM)-F12 containing 10% heat-inactivated fetal bovine serum, endothelial cell growth supplement (15 μg/mL), insulin transferrin selenium (1%), Glutamax (1%) and antibiotic/antimycotic (1%) in an environment of 95% O_2_ and 5% CO_2,_ as described previously.^17^ Cells from the sixth to eighth passage were incubated in 5-mM or 20-mM D-glucose (glucose) for 6 to 96 hours in the presence or absence of inhibitors of Dnmts (5-deoxy-2-Azacytidine, 5-Aza, 1 μM; catalog no. A3656; Sigma-Aldrich Corp., St. Louis, MO, USA) or Tets (2-hydroxyglutarate, 2-HG, 500 μM; catalog no. 16374; Cayman, Ann Arbor, MI, USA).^9,18^ The role of p65 in *Rac1* transcriptional activation was confirmed in the cells incubated with a cell-permeable inhibitor of NF-κ B activation, SN 50 (50 μg/mL; Biomol Research Laboratories, Plymouth Meeting, PA, USA).^19^ A group of cells was transfected with a single siRNA pool of *Dnmt1* or *Tet2* (Cat. Nos. sc-35204 and sc-88934, respectively; Santa Cruz Biotechnology, Santa Cruz, CA, USA) using siRNA transfection reagent (catalog no. sc-29528; Santa Cruz Biotechnology), and the transfected cells were incubated in 5-mM or 20-mM glucose for 96 hours.^9^ The transfection efficiency was evaluated by quantifying mRNA levels of the respective genes (*Dnmt1* or *Tet2*) by SYBR green–based real-time quantitative reverse transcription PCR (qRT-PCR) and by protein expression (western blotting), and it was ∼40% for *Dnmt1*-siRNA and >50% for *Tet2-*siRNA. Untransfected cells and cells transfected with *Dnmt1*-siRNA or *Tet2-*siRNA had similar *Dnmt3a* or *Tet1* expression, confirming the specificity of the *Dnmt1-* and *Tet2-* siRNA. (Fig. 1). Parallel controls included HRECs incubated in 20-mM L-glucose (osmotic/metabolic), and cells transfected with scrambled RNA (transfection).

### Mice

Seven- to 8-week-old C57BL/6J mice (either sex) were made diabetic by streptozotocin injection (55 mg/kg body weight for 4 consecutive days). Mice with blood glucose more than 250 mg/dL 2 days after the last injection were considered diabetic.^20^ Soon after establishment of diabetes, a group of diabetic mice received intravitreal administration of 2-μg *Dnmt1*-siRNA (ID: MSS203624; Thermo Fisher Scientific, Waltham, MA, USA); this stealth *Dnmt1* siRNA is a combination of sequences targeting three positions at exon 4. *Dnmt1*-siRNA was suspended in 2-μL nuclease-free water and mixed with Invivofectamine (catalog no. IVF 3001; Invitrogen, Carlsbad, CA, USA). The right eye received *Dnmt1*-siRNA and the left eye received a medium GC content negative control siRNA (catalog no. 12935-300; Thermo Fisher Scientific). Mice were anesthetized by intraperitoneal injection of ketamine/xylazine (100 mg/kg ketanest and 12 mg/kg xylazine), and using a 32-gauge needle attached to a 5-μL glass syringe (Hamilton), intravitreal injections were performed under a dissecting microscope. Care was taken to position the needle 1 mm posterior to the limbus, and siRNA mixture was slowly injected into the vitreous chamber.^21^ Four weeks after administration of siRNA, the mice were killed, and the retina was quickly isolated. Age-matched normal mice and diabetic mice, without receiving any siRNA, served as controls. The treatment of animals conformed to the ARVO Statement for the Use of Animals in Ophthalmic and Vision Research, and was approved by the Wayne State University's Institutional Animal Care and Use Committee.

Freshly isolated retina was used to prepare microvessels by incubating it in 5-mL de-ionized water for 60 minutes in a shaking water bath at 37°C. The nonvascular tissue was gently removed under the microscope.^6^

### Quantification of 5mC and 5hmC

Genomic DNA was isolated using Qiagen DNA isolation kit (Qiagen, Valencia, CA, USA), and was immunoprecipitated with 5mC or 5hmC antibodies. The levels of 5mC and 5hmC were quantified using methylated or hydroxymethyldated DNA Immunoprecipitation (MeDIP)/(hMeDIP) kits, respectively (Cat. Nos. P-1015 and P-1038, respectively; EPIGENTEK, Farmingdale, NY, USA). The enrichment of 5mC or 5hmC at the *Rac1* promoter was analyzed by qRT-PCR using specific primers.^9^

### Chromatin Immunoprecipitation (ChIP)

The binding of DNA modifying enzymes, or of the transcription factor NF-κB (p65 subunit), at the *Rac1* promoter was determined by ChIP assay using cross-linked samples sonicated in ChIP lysis buffer. Protein-DNA complex (100 μg) was immunoprecipitated with Dnmt1, Tet2, or p65 antibody (Cat. Nos. ab13537, ab135087, and ab7970, respectively; Abcam, Cambridge, MA, USA). Each experiment included antibody control, where the samples were immunoprecipitated with IgG (catalog no. ab171870, Abcam). The immunoprecipitated complex was captured using Protein A Agarose/Salmon Sperm DNA (catalog no. 16157; EMD Millipore, Temecula, CA, USA), washed and de-crosslinked at 65°C for 6 hours, followed by DNA isolation with phenol:choloroform:isoamylalcohol, using the methods reported previously.^8^ Relative binding of Dnmt1/Tet2/p65 at the promoter was quantified by qRT-PCR using *Rac1* promoter specific primer (Table). The target values were normalized to the input controls respectively to obtain fold change.

**Table:**
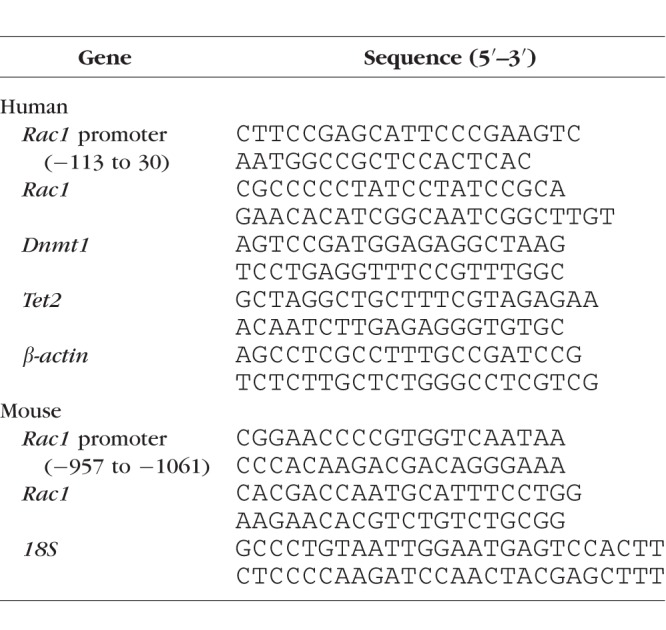
Primer Sequences

### Gene Transcripts

Gene expression was quantified using gene/species-specific primers by qRT-PCR (Table), and the specific products were confirmed by SYBR green single melt curve analysis. The results were normalized to the expression of the housekeeping gene *β-actin* (human) or *18S* (mice), and the relative fold change was calculated using delta delta Ct method.^8^

### Rac1 Activation

Active Rac1 was measured by G-LISA colorimetric assay kit (catalog no. BK-128; Cytoskeleton, Denver, CO, USA) in 20- to 25-μg protein, as described previously.^2^ Fold change was calculated considering the values obtained from cells in normal glucose as one.

### Reactive Oxygen Species

Total ROS levels were quantified using 2′,7′-dichlorofluorescein diacetate (DCFH-DA; catalog no. D6883; Sigma-Aldrich Corp.). Briefly, 5-μg protein was incubated with 4-μM DCFH-DA for 20 minutes, and the fluorescence was measured at 485-nm excitation wavelength and 535-nm emission wavelengths. Fold change was calculated as percent change with respect to values obtained from cells in normal glucose.^22^

### Statistical Analysis

Data are presented as mean ± SD. Comparison between groups was made using 1-way ANOVA followed by Dunn's *t*-test; *P* < 0.05 was considered significant.

## Results

Rac1 is activated in hyperglycemic milieu in the retina and its vasculature and its gene transcripts are increased^2,22^; to investigate the role of DNA methylation in *Rac1* transcriptional regulation, 5mC levels at the *Rac1* promoter were quantified in the cells exposed to high glucose for 96 hours, a duration when cell apoptosis is detected.^23^ Figure 1a shows that high glucose decreased 5mC levels by 50%. Because 5mC is associated with transcriptional repression,^24^ and hyperglycemic milieu activates both Dnmts and Tets,^9^ the effect of high glucose on hydroxymethylation of 5mC was investigated; compared with cells in normal glucose, cells incubated in high glucose had 2-fold higher 5hmC levels at *Rac1* promoter (Fig. 1b).

**Figure 2:**
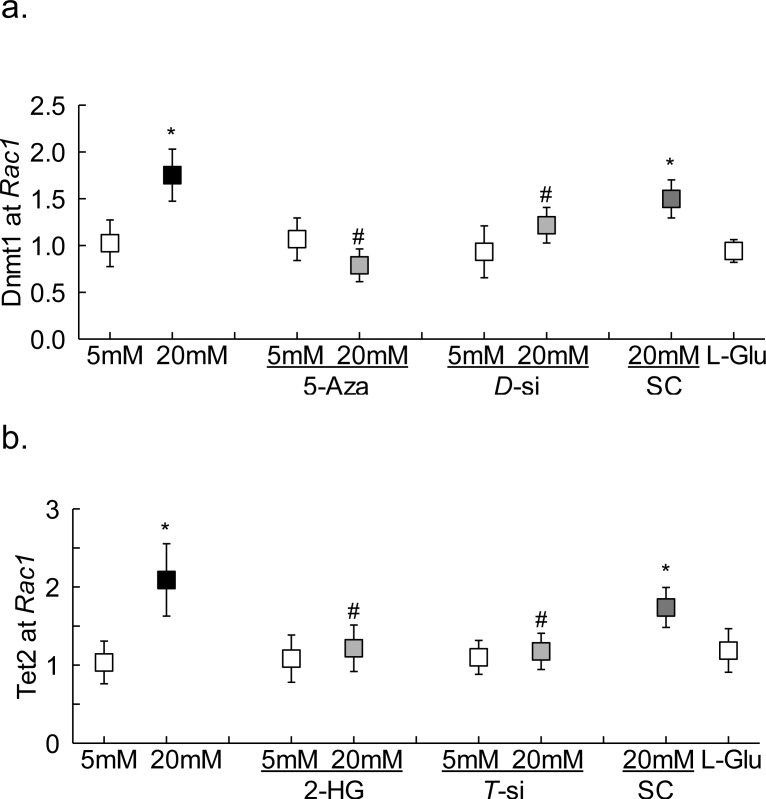
Dnmt1 and Tet2 binding at Rac1 promoter. The binding of (a) Dnmt1 or (b) Tet2 was determined by ChIP technique in cells incubated in high glucose for 96 hours using IgG as an antibody control. Measurements were made in duplicate in three to four different cell preparations. The values obtained from cells in 5-mM glucose are considered as 1. *P < 0.05 compared with 5-mM glucose; #P < 0.05 compared with 20-mM glucose.

To investigate the mechanism responsible for decrease in 5mC at *Rac1* promoter, despite activation of Dnmts, in hyperglycemic milieu,^9^ the binding of Dnmt1 was investigated. Compared with the cells in normal glucose, high glucose exposure for 96 hours increased Dnmt1 binding by more than 1.5-fold, and in the same samples, Tet2 binding at *Rac1* promoter was also elevated by more than 2-fold (Figs. 2a, 2b).

To confirm the role of active DNA methylation-hydroxymethylation in the regulation of *Rac1* promoter methylation status, the effect of inhibition of Dnmt by its chemical (5-Aza) or molecular (*Dnmt1*-siRNA) inhibitors was analyzed; both 5-Aza and *Dnmt1*-siRNA prevented glucose-induced increase in Dnmt1 binding at *Rac1* promoter (Fig. 2a). However, these inhibitors had no effect on Dnmt1 binding in the normal glucose conditions. To further understand the role of 5mC hydroxymethylation in *Rac1* transcriptional regulation, the effect of Tet regulation on its binding at *Rac1* promoter was investigated. Figure 2b shows that Tet2 inhibition either by 2-HG or *Tet2*- siRNA significantly attenuated glucose-induced increase in Tet2 binding. In the same cell preparations, inhibition of Dnmts or Tets also ameliorated glucose-induced increase in 5hmC levels at *Rac1* promoter (Fig. 1b). Untransfected cells or scrambled RNA transfected cells, incubated in high glucose for 96 hours, had similar values, and also values from cells in 20-mM L-glucose or in normal glucose were not different from each other, confirming the use of 20-mM L-glucose as a valid osmotic/metabolic control.

**Figure 3:**
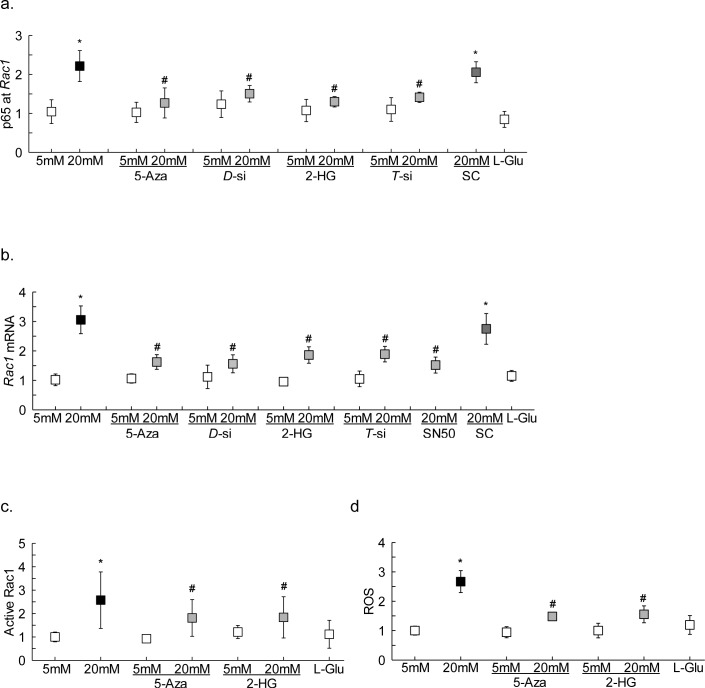
Effect of regulation of DNA methylation/hydroxymethylation on transcription factor binding and Rac1. HRECs incubated in high glucose for 96 hours were analyzed for (a) p65 binding at Rac1 promoter by ChIP technique. Rac1 (b) gene transcripts were quantified by qRT-PCR and (c) activation by G-LISA. (d) ROS levels were measured by DCFH-DA. Measurements were made in duplicate in three different cell preparations. The values obtained from cells in 5-mM glucose are considered as 1, and are represented as mean ± SD; 5 mM and 20 mM indicate 5-mM and 20-mM glucose, respectively; 5-Aza, 2-HG, and SN50 indicate cells incubated in 20-mM glucose with 5-Aza or 2-HG or SN50; D-si, T-si, and SC indicate Dnmt1-siRNA or Tet2-siRNA or scrambled control RNA, respectively; and L-Glu indicates 20-mM L-glucose.

Inhibition of DNA methylation-hydroxymethylation machinery also attenuated glucose-induced increase in p65 binding at *Rac1* promoter, and the values obtained from cells in high glucose with Dnmt or Tet inhibitors were not significantly different from the cells in normal glucose (Fig. 3a). Consistent with the p65 binding, these inhibitors ameliorated glucose-induced increase in Rac1 gene transcripts and activity, and ROS levels (Figs. 3b–d). The role of NF-κB in *Rac1* transcriptional activation was confirmed by inhibiting glucose-induced NF-κB activation by SN50, and as shown in Figure 3b, SN50 ameliorated glucose-induced increase in *Rac1* transcription.

**Figure 4:**
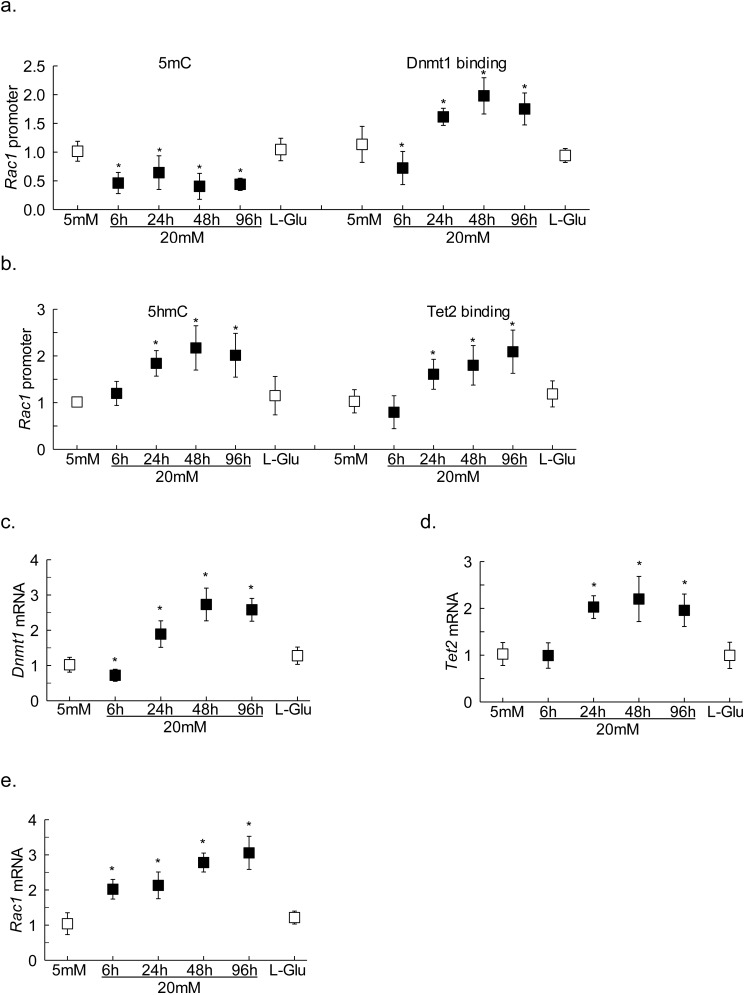
Temporal relationship between promoter DNA methylation and transcription of Rac1. Cells incubated in 5-mM or 20-mM glucose for 6 to 96 hours were analyzed for (a) 5mC levels and Dnmt1 binding and (b) 5hmC levels and Tet2 binding. Gene transcripts of (c) Dnmt1, (d) Tet2, and (e) Rac1 were measured by qRT-PCR. Values obtained from cells in 5-mM glucose are considered as 1, and are represented as mean ± SD from three different cell preparations, each measurement made in duplicate; 5 mM and 20 mM indicate 5-mM and 20-mM glucose, respectively; L-Glu indicates 20-mM L-Glucose. *P < 0.05 compared with 5-mM glucose.

Rac1-Nox2-ROS signaling is one of the early events in the pathogenesis of diabetic retinopathy; in retinal endothelial cells it can be seen within 6 hours of high glucose insult.^2^ To investigate if DNA-methylation-hydroxymethylation is playing any role in the early activation of *Rac1*, DNA methylation status of *Rac1* promoter was analyzed in HRECs exposed to high glucose for 6 to 48 hours. Compared with cells in normal glucose, while at 6 hours of high glucose both 5mC levels and Dnmt1 binding were decreased, at 24 hours and beyond, despite increased Dnmt1 binding, 5mC levels remained subnormal (Fig. 4a). Consistent with this, although *Dnmt1* transcripts were elevated by approximately 2-fold at 24 hours (and beyond) of exposure of high glucose, at 6 hours *Dnmt1* expression was significantly decreased (Fig. 4b). In the same samples, 5hmC levels at *Rac1* promoter were, however, not affected, but within 24 hours, >50% increase in 5hmC was observed. In accordance, 6 hours of high glucose also failed to affect Tet2 binding at *Rac1* promoter, and the gene transcripts of *Tet2* were also not different from those obtained from cells in normal glucose. But, at 24 hours (and beyond) of high glucose incubation, 5hmC levels, Tet2 binding, and its gene transcripts were significantly elevated, and they remained elevated at 48 to 96 hours of high glucose exposure (Figs. 4c, 4d). Cells in high glucose for 6 hours and beyond, as expected,^6,22^ had an approximately 2-fold increase in *Rac1* gene transcripts (Fig. 4e). Values obtained from cells in 20-mM L-glucose were not different from those obtained from cells in 5-mM glucose.

**Figure 5:**
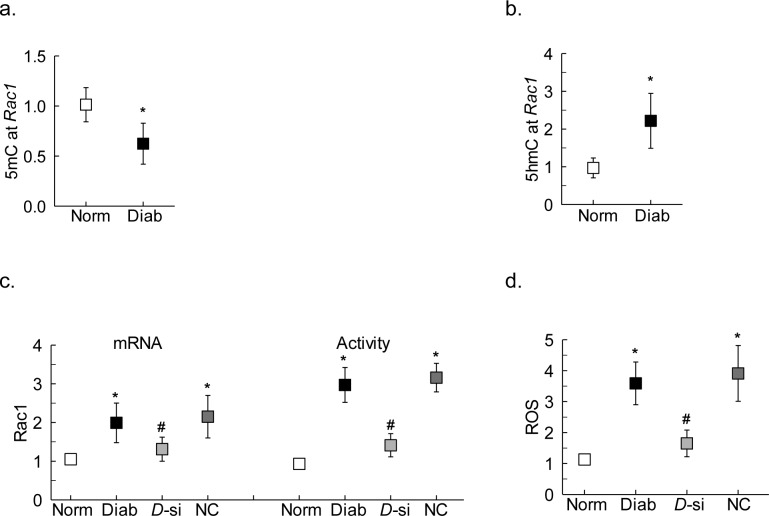
Effect of diabetes on Rac1 promoter DNA methylation. Retinal microvessels from diabetic mice were analyzed for (a) 5mC and (b) 5hmC levels using IgG as an antibody control. Retinal microvessels from the eye of diabetic mice receiving intravitreal administration of Dnmt1-siRNA or negative control siRNA were analyzed for (c) Rac1 gene transcripts and activation, and (d) ROS levels. Values are represented as mean ± SD from five to six mice in each group. Norm and Diab indicate normal and diabetic mice, respectively; D-si and NC indicate retinal microvessels from the eye of diabetic mice receiving Dnmt1-siRNA or negative control siRNA, respectively. *P < 0.05 compared with normal; #P < 0.05 compared with diabetes or NC.

To confirm the key parameters in an in vivo model, DNA methylation status of the *Rac1* promoter was investigated in retinal microvessels obtained from diabetic mice. Compared with normal mice, although diabetic mice had decreased 5mC levels at the *Rac1* promoter, 5hmC levels were significantly elevated (Figs. 5a, 5b). The role of DNA methylation in *Rac1* transcriptional regulation was further confirmed in the mice receiving intravitreal administration of *Dnmt1*-siRNA. Although due to some technical issues, *Dnmt1* silencing could not be confirmed in the target cells, and off-target effects of the *Dnmt1-*siRNA could not be investigated, administration of siRNA ameliorated diabetes-induced increase in gene transcripts and activity of Rac1 and ROS levels; the values obtained from the retinal microvessels prepared from the diabetic mouse eye receiving *Dnmt1*-siRNA were significantly different from those obtained from the eye receiving siRNA negative control (Figs. 5c, 5d).

## Discussion

Diabetic retinopathy has a complex underlying pathophysiology, and in spite of cutting edge research in the field, molecular events contributing to its development remain unclear. Among many abnormalities implicated in the development of diabetic retinopathy, experimental models have documented oxidative stress to be at a central place.^1^ Although mitochondria are the major source of ROS, ROS are also produced in the cytosol by Nox enzymes,^25^ and our previous study has shown that in the pathogenesis of diabetic retinopathy, activation of cytosolic Nox2 precedes mitochondrial damage.^2,5^ Rac1, an obligatory component of the multicomponent Nox2, is activated in hyperglycemic milieu in the retina and its vasculature, and its gene transcripts are elevated.^2,6,22^ The data provided in the current report suggest the role of epigenetic modifications in *Rac1* transcriptional regulation. DNA at the *Rac1* promoter undergoes active methylation-hydroxymethylation; in the initial stages of hyperglycemic insult (6 hours of high glucose), due to inhibition of Dnmts, the promoter is hypomethylated. However, at 24 hours, although the binding of Dnmt1 is increased, concomitant activation of Tets hydroxymethylates 5mC to 5hmC, resulting in *Rac1* activation. Role of active DNA methylation-hydroxymethylation in regulation of *Rac1* transcription is further confirmed by our results showing increased 5hmC levels at the *Rac1* promoter in the retinal microvessels from mice diabetic for 2 weeks, and amelioration of diabetes-induced activation of Rac1 (transcription and activity) by *Dnmt1*-siRNA. These results have significant implications for diabetic retinopathy because, although the appearance of retinal histopathology, characteristic of diabetic retinopathy, in rodent models is visible only after 6 to 8 months of diabetes, Rac1-Nox2-ROS signaling is an early event, which can be seen within 2 weeks after induction of diabetes.

Addition of a methyl group to carbon-5 of a cytosine base, forming 5mC, is considered as a fundamental epigenetic mediator, and is an important component in many cellular processes, including genomic imprinting and chromosome stability.^26^ DNA methylation is mainly associated with repression of gene expression by changing the chromatin structure and restricting access of the transcription factors to the gene promoter.^24^ However, errors in DNA methylation are observed with many genes in chronic diseases including cancer and diabetes.^9,27–29^ Human *Rac1* promoter has four CpG islands within −1200 bases upstream of transcription start site, and the binding site for the transcription factors lies in the CpG island close to the transcription start site between −317 to 147 bp.^30^ Our previous work has shown that in diabetes, the binding of transcriptional factor NF-κB at *Rac1* promoter is increased in the retinal vasculature,^6^ and here we show that at a duration of high glucose insult when mitochondrial damage and apoptosis are also observed (96 hours), 5mC levels are also decreased. As mentioned above, Dnmts catalyze the transfer of a methylation residue from S-adenosyl-L-methionine to cytosine, and among these, Dnmt3a and Dnmt3b are the de novo methyltransferases, Dnmt1 is considered as the maintenance methyltransferase and recognizes hemimethylated sites restoring symmetric methylation during replication.^31^ In diabetes, Dnmts are activated, and among this family of enzymes, only Dnmt1 is upregulated in the retina and its vasculature.^9^ At 96 hours, despite decrease in 5mC levels, binding of Dnmt1 at *Rac1* promoter is increased, suggesting that the 5mC formed by Dnmt1 could be demethylated. The results from the in vivo model further support the role of active DNA methylation in *Rac1* activation, as administration of *Dnmt1*-siRNA ameliorates both *Rac1* transcription and activation, and decreases ROS levels, seen in the retinal microvasculature of diabetic rodents.

DNA is a dynamic structure, and the methylated cytosine can be rapidly demethylated.^13^ Demethylation of cytosine is considered necessary for epigenetic reprogramming of genes, and is directly involved in many diseases including tumor progression.^14^ Demethylation of DNA can either be passive or active, or a combination of both, and the enzyme-based oxidation of 5mC to 5hmC promotes DNA demethylation by binding to CpG-rich regions to prevent unwanted Dnmt activity.^13^ Retinal Tets are activated in diabetes, and among this family of enzymes, Tet2 is the only member with upregulated gene transcripts.^9^ The results presented here clearly show that, although Dnmt1 binding at *Rac1* promoter is increased in diabetes, due to increased Tet2 binding, 5hmC levels are elevated, suggesting a rapid hydroxymethylation of 5mC formed by Dnmt1. This increase in 5hmC allows binding of the transcription factor, increasing *Rac1* expression. Further, we show that inhibition of Tets, in addition to regulating DNA methylation status of *Rac1* promoter, also inhibits NF-κB binding and *Rac1* transcription, further confirming the role of active DNA methylation in *Rac1* activation. In support, diabetes modulates transcription of matrix metalloproteinase *MMP-9*, an enzyme implicated in mitochondrial damage, via dynamic DNA methylation-hydroxymethylation of its promoter.^9^ Moreover, hyperglycemia has a direct impact on changes in the epigenome, and high DNA methylation and hydroxymethylation levels are observed in poorly controlled diabetic patients compared with healthy individuals.^32^ We recognize that Tets can further oxidize 5hmC to 5-formylcytosine, and 5-formylcytosine to 5-carboxylcytosine^33^; their role in regulation of *Rac1* transcription cannot be ruled out. Although methylated CpGs are considered to influence transcription factor binding at their recognition site, and DNA methylation at promoter generally locks genes in a stable silent state, many transcription factor recognition sites do not possess CpGs in their recognition sequence,^10^ and their role in maintaining *Rac1* gene transcription remains to be examined. However, amelioration of diabetes-induced increased NF-κB binding at the *Rac1* promoter by both Dnmt and Tet inhibitors, observed in the present study, clearly suggests that dynamic CpG methylation is playing a critical role in the regulation of binding of this transcription factor.

Diabetic retinopathy is a progressive disease, and activation of Rac1-Nox2 is one of the early events in its pathogenesis. Rac1-Nox2–mediated cytosolic ROS play a major role in damaging the mitochondria, leading to the development of histopathology characteristic of diabetic retinopathy.^2^ DNA methylation varies in a global and local context, and active changes in DNA methylation are observed during aging.^34–37^ Site-specific hypermethylation are observed predominantly in promoter regions,^38,39^ and methylation marks are also considered to predict the age of the organism.^37,40^ Our data show that in the early stages of diabetic retinopathy (6 hours of high glucose exposure), although *Tet2* is not affected, *Dnmt1* is repressed, and promoter DNA is hypomethylated, resulting in *Rac1* transcriptional activation. However, as the disease progresses, the two enzymes with opposing functions, Dnmts and Tets, are both activated. Although there could be increase in 5mC at the promoter, activated Tets overpower Dnmts activation, and hydroxymethylate 5mC to 5hmC, transcriptionally activating *Rac1*.

There are some limitations of our study, including the following:

- Retinal endothelial cells are not the only cells that are lost in the early stages of diabetic retinopathy; instead, pericytes of the vasculature also undergo accelerated apoptosis before any pathology, characteristic of diabetic retinopathy, can be observed, and in diabetes, photoreceptors are also a major source of ROS.^41–43^ The possibility of a similar mechanism(s) operating in retinal pericytes, and in other nonvascular cells, cannot be ruled out.
- Diabetic retinopathy is a slow progressing disease, mitochondrial damage and capillary cell apoptosis precedes the development of microvascular histopathology, and the histopathology not visible until 6 to 8 months of diabetes in rodents,^23,42,44,45^ raising a possibility that the in vitro model might not fully reproduce the in vivo model. However, most of the mechanism-based data are comparable between the in vitro and in vivo models.^2,5,9,17^
- As mentioned above, high glucose-induced increase in Rac1-ROS can be seen within 6 hours in retinal endothelial cells and within 15 days of diabetes in rodents, and increase in mitochondrial damage and capillary cell apoptosis within 96 hours in cells and approximately 6 months of diabetes in mice. However, our retinal microvessel Rac1-ROS data, presented here, are from diabetic mice receiving *Dnmt1*-siRNA for only 4 weeks. In support of a possible link between early changes in DNA methylation and the onset of diabetic retinopathy, our previous work has shown that inhibition of the enzymes responsible for maintaining DNA methylation ameliorates mitochondrial damage and capillary cell apoptosis, the events that precede retinal microvascular pathology in the in vivo models of diabetic retinopathy.^9,46^
- Because epigenetic modifications are influenced by external factors, and in vitro culture of endothelial cells may introduce additional epigenetic modifications, this may interfere with the true recapitulation of the disease state. To alleviate the effect of cell passaging, the present study compared *Rac1* promoter DNA methylation in high glucose and normal glucose conditions using HRECs from the same passage (and the same batch). Moreover, experiments in in vitro models for many chronic diseases. including diabetic retinopathy, using appropriate controls, have provided consistent results as seen in the in vivo disease models.^8,10,47,48^

**Figure 6:**
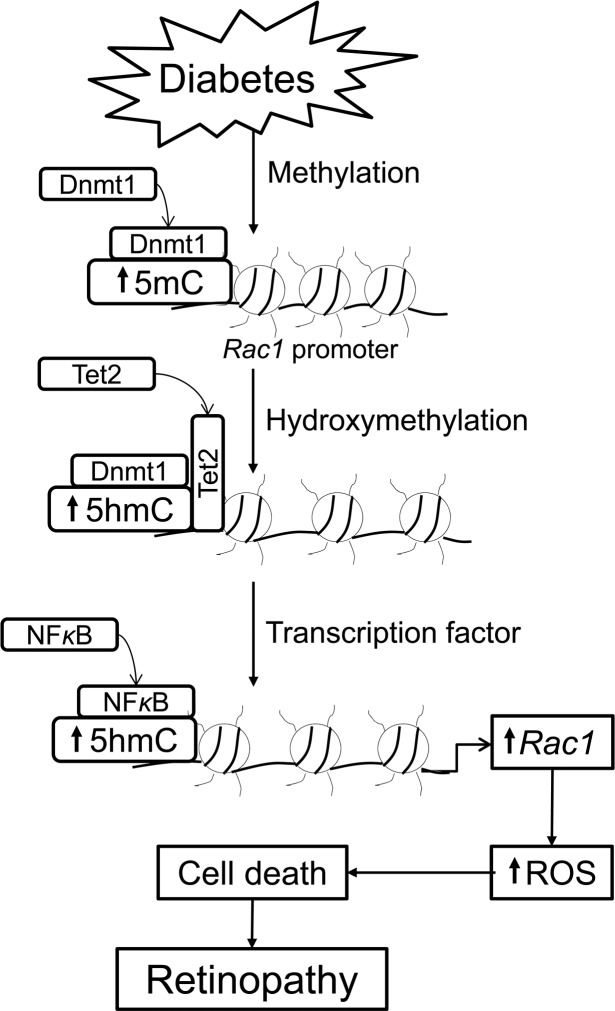
Graphic presentation. Diabetes increases Dnmt1, and its increased binding at the Rac1 promoter results in 5mC formation. Concomitant increase in Tet2, however, converts 5mC to 5hmC, which facilitates transcription factor binding. Activated Rac1, via Nox2 signaling, elevates cytosolic ROS levels, and ROS, via damaging mitochondria, increase capillary cell apoptosis, ultimately resulting in diabetic retinopathy.

In summary, despite the limitations stated above, our convincing data clearly demonstrate a role of active DNA methylation-demethylation in the regulation of *Rac1* in the development of diabetic retinopathy. Although DNA methylation is an epigenetic repressive mark, hydroxymethylation of 5mC is also now considered as an independent epigenetic marker; imbalance in genomic 5mC/5hmC levels are associated with aberrant gene activation and oncogenic transformation.^49,50^ Thus, despite increased 5mC formed at the *Rac1* promoter due to activation of Dnmt1, concomitant increase in Tet2 hydroxymethylate it to 5hmC. This allows the transcription factor to bind, and activate *Rac1* transcription. Rac1, via Nox2, elevates cytosolic ROS levels, and ROS damage mitochondria and increase capillary cell apoptosis, which, ultimately, results in diabetic retinopathy (Fig. 6). These data clearly imply a critical role of DNA methylation in diabetic retinopathy, and demonstrate the importance of DNA methylation/hydroxymethylation machinery in regulation of Rac1-mediated oxidative stress. This opens up the possibility of using this machinery as a target to regulate ROS production during early stages of the disease, which could help diabetic patients from facing the devastating consequences of this progressing disease, and protect them from losing their vision.
